# Dentist Involvement in the Treatment of Radiation-Induced Oral Mucositis—A Pilot Cross-Sectional Study

**DOI:** 10.3390/dj12050134

**Published:** 2024-05-08

**Authors:** Monika Burja Vladić, Ana Andabak-Rogulj, Krešimir Gršić, Vlaho Brailo, Božana Lončar Brzak, Ivana Škrinjar, Danica Vidović Juras

**Affiliations:** 1 Department of Oral Medicine, University of Zagreb School of Dental Medicine, 10000 Zagreb, Croatia; mburja@sfzg.hr (M.B.V.); loncar@sfzg.hr (B.L.B.); 2 Department of Oral Medicine, University of Zagreb School of Dental Medicine, University Hospital Center Zagreb, 10000 Zagreb, Croatia; brailo@sfzg.hr (V.B.); iskrinjar@sfzg.hr (I.Š.); djuras@sfzg.hr (D.V.J.); 3 Department of Otorhinolaryngology and Head and Neck Surgery, University Hospital Center Zagreb, 10000 Zagreb, Croatia; kresimir.grsic@yahoo.com

**Keywords:** head and neck cancer, oral cancer, radiotherapy, oral mucositis

## Abstract

**Objectives:** Oral mucositis (OM) occurs in more than 95% of patients irradiated in the head and neck area. This paper aims to determine the occurrence and characteristics of OM in patients with head and neck cancer (HNC), as well as the involvement of dentists/oral medicine specialists in treating such patients. **Methods**: This study was conducted at the Department of Otorhinolaryngology and Department of Oral Medicine, University Hospital Center Zagreb, from April to August 2022, on patients irradiated in the head and neck area. A unique OM questionnaire was created on the incidence, characteristics, oral care, and involvement of dentists in the overall care. **Results**: Thirty patients filled out the questionnaire. Of the 22 patients who had developed OM, 14 had grade-three OM. Ten patients were treated for OM in line with the instructions of an oral medicine specialist, eight based on the instructions of a specialist responsible for monitoring of the underlying disease, and four were not treated at all. Sixteen patients had not been referred to a dentist before the start of RT. **Conclusions**: These results showed insufficient care and treatment of OM, as well as insufficient involvement of dentists in the oncology team.

## 1. Introduction

Head and neck cancers (HNCs) are the seventh most common type of cancer in the world. In 2020, more than 900,000 new cases were reported, according to Global Cancer Statistics, of which 158,581 cases were diagnosed in Europe [1]. In the same year, 1000 cases of HNC were diagnosed in Croatia, of which 476 died [2]. The incidence of this group of cancers is growing rapidly; therefore, an increase of as much as 30% is expected by 2030 (1.08 million annually worldwide) [2]. Malignant tumors of the head and neck area include the oral cavity, oropharynx, nasopharynx, hypopharynx, larynx, salivary glands, nasal cavity, and sinuses [2]. Oral cancer (OC) is the most serious diagnosis seen in dental practice and most often occurs in men over 40 years of age who consume tobacco and alcohol; however, recently, an increasing incidence has been noted in women, which is attributed to a change in lifestyle habits. The average age of OC occurrence is 60 years [3]. 

Tobacco products are considered to be the main risk factor for the development of HNC. According to some research from the literature, almost 90% of OC cases in men are associated with the excessive use of cigarettes, while women account for 60% of cases. To date, more than 70 known carcinogens in cigarette smoke have been detected. Harmful tobacco compounds such as nitrosamines and polycyclic aromatic hydrocarbons penetrate mucous membrane, bind to cellular DNA, and consequently cause its damage [4]. Alcohol is the second most common cause of HNC. Acetaldehyde prevents the synthesis and repair of DNA, and ethanol is a solvent for carcinogens from tobacco smoke, which facilitates their passage into the oral mucosa. The latter theory could explain the synergistic effect of the joint consumption of alcohol and cigarettes [5]. Furthermore, in the group of HNCs, the highest frequency of human papillomavirus (HPV) is found in the oropharynx, but its role in the development of OC is still controversial [6]. In addition, other factors, such as chronic irritation, malnutrition, genetic factors, and carcinogenic air pollutants, also affect the development of HNC. Foods specific to the region of India, Asia, and South America, such as betel nuts and Mate tea, are associated with the occurrence of oral cancer (OC) [7,8].

OC, which primarily occurs on the soft tissues of the oral cavity, the so-called risk areas—lateral and ventral sides of the tongue, the floor of the mouth, and buccal mucosa—is of particular interest to dentists [7]. In its earliest stages, OC is almost entirely curable with surgery. Unfortunately, in the earliest stages, cancer is asymptomatic or shows virtually no symptoms. More than 60% of patients get examined at a later stage, when, in addition to surgical removal of the cancer, radiotherapy (RT) or adjuvant chemotherapy (CT) and RT are also required. Radiotherapy to the head and neck area leaves numerous side effects in the oral cavity. The first side effect which occurs within ten days of starting RT is oral mucositis (OM). Oral mucositis is an ulcerative inflammation of the mucous membrane. More severe forms disrupt the daily functioning of the patient, making it impossible to eat and speak. OM is probably the most difficult side effect to tolerate, which can often lead to the interruption of RT and compromise the healing process due to intolerable symptoms. It should be pointed out that every day without RT reduces the survival rate by 1% [9,10,11,12,13]. Many scales are used for grading OM. This study uses the World Health Organization (WHO) scale, which incorporates four degrees of mucositis, depending on the clinical picture and the ability to consume food: I (mild)—erythema and edema of the mucosa; II (moderate)–erythema and ulcerations, and the patient can eat solid food; III (severe)–ulcerations, and the patient can take liquids but not solid foods; and IV (life-threatening)—the patient is on parenteral nutrition [12].

The prevention and treatment of OM both remain challenging; therefore, the treatment is mainly symptomatic. The Multinational Association of Supportive Care in Cancer/International Society of Oral Oncology (MASCC/ISOO) guidelines for the treatment of OM suggest several treatment modalities such as benzydamine hydrochloride, polyvinylpyrrolidone, topical lidocaine, photobiomodulation, cryotherapy, natural remedies such as turmeric or honey [13]. 

The hypotheses of this research are that more attention should be paid to the treatment of OM and it should be treated in accordance with the relevant guidelines, and that doctors of dental medicine are often not included in the oncology team when treating patients with HNC. This research aims to collect and analyze data on the experience of OM in patients treated with HNC radiation, its frequency, characteristics, treatment method, oral care, and the involvement of dentists in the overall care, while comparing the results of this study with data from relevant literature.

## 2. Materials and Methods

### 2.1. Study Design

This research was approved by the Ethics Committee of the School of Dental Medicine, University of Zagreb, under number 05-PA-30-V-2/2022. After the examination, the patients received a questionnaire that they voluntarily filled out. Before filling out the questionnaire, the purpose of the research was explained, and an informed-consent form was issued. By signing the informed consent form, the respondent accepted participation in the research. Completing the questionnaire was voluntary, and the respondent could withdraw from the research even after filling out the form.

### 2.2. Participants

This study included 30 patients irradiated in the head and neck area. All the participants were adults in regular follow-up examinations from April to August 2022 at the Department of Otorhinolaryngology and Department of Oral Medicine, University Hospital Center Zagreb. Inclusion and exclusion criteria for the participants are presented in Table 1.

### 2.3. Questionnaire 

The investigators (DVJ and MBV) created a unique questionnaire for this research according to the data they considered important. The questionnaire consists of 46 questions, but only 27 questions directly or indirectly related to OM were selected for analysis in this study. The questionnaire was initially written in Croatian, but for the purpose of this paper, it was translated into English.

### 2.4. Statistics 

The data were entered into a Microsoft Excel table and were presented with methods of descriptive statistics using absolute and cumulative frequencies. Statistical processing analysis was performed in the IBM SPSS 25 program (IBM Corp, Armonk, NY, USA).

## 3. Results

The patients were mostly men, older than 50 years, and the most common localizations of cancer were the tongue and oropharynx (Table 2). 

In 22 cases, the specialist otorhinolaryngologist was the first doctor to discover the malignant change; in 6 cases, the dentist suspected a malignant change; and in 2 cases, the general practitioner. Before contacting the doctor, most patients were aware of changes for a period of one to several months, while only eight were aware of the changes for a period of less than a month. Patients usually waited a week or two for the first specialist examination. Most patients waited two months for the start of RT (Table 3).

Slightly more than half of the respondents, 16 of them to be exact, had not been referred to a dentist before the start of radiation. All referred respondents visited a dentist after a referral from an otorhinolaryngologist. The largest number of respondents were warned about the possible side effects of radiation. Most respondents were warned about all the consequences of radiation on the oral cavity (Table 4). 

The largest number of patients was irradiated 30 times. Of the total number of 30 patients, 22 developed OM. The largest number of patients developed OM within ten days of starting RT, and in most patients, OM continued for at least two weeks after ceasing RT (Table 5). 

The most common grade of OM in patients who were irradiated alone or in combination with CT was grade three (Table 6).

The patients’ OM was alleviated according to the instructions of dentists or oral medicine specialists and otorhinolaryngologists. The preparation based on polyvinyl-pyrrolidine (Gelclair^®^) was most often used alone or in combination with other preparations (Table 7). 

## 4. Discussion

The largest number of our respondents were middle-aged-to-elderly men, which correlates with several studies showing that the incidence of cancer in the head and neck area is more common in men than in women [14,15,16]. Until recently, it was believed that men are more prone to OC due to their more frequent consumption of alcohol and cigarettes [17]. A ten-year study conducted by Park et al. [18] suggests that this is not exclusively the case. The researchers mentioned above included over 10 million people. During that period, 10,732 people were diagnosed with OC, of which 8500 were men, regardless of whether they consumed large amounts of alcohol or smoked a lot. Further studies are necessary to elucidate the cause of the greater susceptibility of men rather than women [18]. 

In the present study, the largest number of respondents, 13 of them, had OC. Given the small number of respondents, the results cannot be compared relevantly, and a study incorporating a larger number of respondents is needed. In Croatia, according to the latest data from 2020, the largest number of patients with HNC fell into the oral cavity category—339 of them [1].

In most cases (n = 22) in this study, an otorhinolaryngologist was the first professional to recognize that the patient was suffering from HNC, while in six cases, the disease was recognized by a dentist. This finding is not surprising given that most of the respondents were recruited at the Department of Otorhinolaryngology, University Hospital Center Zagreb, and a smaller proportion at the Department of Oral Medicine. This approach to recruiting research subjects certainly affects the results, and the stated conclusions refer only to the involved patients. Further research incorporating a larger number of subjects and a different way of recruiting subjects is needed to obtain more representative results. Although the diagnosis of OC mostly depends on the institution to which patient primarily present their ailments, dentists are in an ideal position to detect cancer early, as they observe and examine oral cavities on a daily basis. In the study by Ligier et al. [19], using a sample of 342 patients, in 21% of cases, the dentist recognized the disease as cancer.

In our study, a majority of patients were already aware of the change in the period of one-to-several months before they decided to consult a doctor. The initial delay in the early diagnosis of OC is mainly related to the so-called patient interval (the time between the first signs and symptoms of OC and consultation with a doctor or dentist). In a study by Kassirian et al. [20], the largest number of HNC patients in Canada was aware of the change for almost four months before seeing a doctor, while in a study by Joshi et al. [21], patients were aware of the change for almost seven months. In their research from 2008, Peacock et al. [22] showed that the mean patient interval was 3.5 months in the US, and similar results were collected in Germany (patient interval, 3–4 months) [23]. 

Research from 2019 [24] shows that patients with OC in China visited a doctor for the first examination one month after the onset of symptoms, and in Iran, after a month and a half (45 days) [25]. In a systematic review, Lima et al. [26] concluded that the largest number of cases of delayed diagnosis is related to patients, mostly due to poor awareness of OC, risk factors, and early signs and symptoms that may indicate cancer. The causes of the delay by health professionals stem from the difficulty in recognizing the lesion, which, in turn, is related to wrong diagnoses, as well as the fact that health professionals are insufficiently informed about OC. 

Radiation therapy, surgery, and CT are the three main treatment modalities for HNC. Chemotherapy is often used as an additional or adjuvant treatment. The optimal combination of three treatments for a patient with a specific HNC depends on the location of the cancer and the stage of the disease. In general, patients with early stage (stage I and II) are treated with a single primary therapy, radiation or surgery, while those with advanced stage (stage III and IV) are often treated with CT and RT, in addition to surgery [27]. The time from the final diagnosis to commencing treatment is called the pre-treatment time. In this study, the largest number of patients waited one-to-two months for surgery and two months for RT. The research of Fujiwara et al. [28] showed that the median pre-treatment time in the US is 30 days, and similar results were obtained by Kaing et al. [29], who conducted a study in Australia. Lyne et al. [30] reported a median pre-treatment time interval of 25 days in Denmark. A study conducted by Tsai et al. [31] showed that patients who waited for treatment longer than 30 days from receiving a diagnosis had a 1.18-to-1.32 times higher risk of death compared to patients treated within 30 days of a diagnosis. The results of a study conducted in the United States of America were similar. Patients who waited for treatment from 61 to 90 days after diagnosis had a higher risk of death compared to patients who were treated within 30 days [32]. A study conducted in the Netherlands also confirms that a longer waiting time is associated with a significantly higher mortality rate [33]. In Brazil, a law was passed in 2012 that stipulates a maximum period of 60 days from diagnosis to cancer treatment for patients [34]. These studies confirm that a prolonged period from the time of diagnosis to commencing treatment can lead to tumor progression, exacerbate treatment, make recovery more difficult, and lead to a lower quality of life.

Although we did not focus on the causes of delays in commencing treatment in this research, which, as mentioned, may be related to reasons directly affecting patients and healthcare organizations, possible causes of delayed initiation of treatment may be related to comorbidities that require the modification of treatment, a lower level of education, and the actual distance of the patient’s place of residence from the treatment facility [35]. Further studies are needed to elucidate the cause-and-effect relationship of later initiation of treatment in Croatia. 

In this study, 14 patients were referred to a dentist by an ENT specialist before commencing RT. Although the research was conducted on a small number of respondents, the fact that all referred patients visited a dentist is encouraging. In the Republic of Croatia, a doctor of dental medicine or an oral medicine specialist is still left out of the official multidisciplinary team. Concerning possible dental problems, patients diagnosed with HNC have a higher prevalence of periodontitis and carious diseases. In addition to remediation of the effects of radiation on the oral cavity, a dentist or oral medicine specialist performs an examination of the oral cavity and hard dental tissues in order to timely detect problematic dental foci, primarily related to periodontal diseases, caries, and pulp diseases [36]. If left untreated, dental problems can lead to the discontinuation of primary treatment and mortality. In addition to treatment interruption, an untreated oral cavity can lead to a more severe form of OM, as well as osteoradionecrosis, a lifelong side effect after radiation to the head and neck area [37].

Bertl et al. [36] showed that patients who were treated by a multidisciplinary team that included a dentist had a better oral status after the end of treatment and, thus, a lower risk of osteoradionecrosis compared to patients who were treated by a team without a dentist, and where the team only suggested visiting a dentist. Dental treatment and good oral hygiene are important factors that reduce the risk of oral and other diseases that can be induced by RT. Therefore, it can be concluded that doctors of dental medicine have an important role in the prevention and treatment of complications in the oral cavity before, during, and after RT [11].

Patients who were not referred to a dentist by a specialist responsible for the underlying disease in this study did not undergo a dental examination or preparation for oncological treatment on their own initiative. This information is not surprising considering that Croatia is among the worst countries in Europe according to the DMFT index (decayed, missing, and filled teeth) based on the most recent research from 2015 [38]. A possible justifying assumption could be a lack of awareness on the importance of a dental visit prior to irradiation treatment, as well as the fact that patients are preoccupied with their primary illness and only contact a dentist if an acute condition occurs. In our study, most patients were irradiated 30 times, meaning that they received a total dose of 60–70 Gy divided into 2 Gy daily fractions (5 days a week) for 6–7 weeks. Most oral complications occur when doses exceeding 45 Gy are applied [32]. The oral side effects of RT are the result of the harmful effect of ionizing radiation and can occur during or after the completion of therapy. Acute side effects are OM (oral mucositis), dysgeusia, and xerostomia, which appear immediately at the beginning of RT [5]. Chronic side effects are trismus, radiation caries, and osteoradionecrosis. The risk of developing these side effects is lifelong [10].

The research shows that 22 patients were warned about the consequences of radiation on the oral cavity—i.e., most (n = 19) were informed about it (OM, dryness, taste disturbance, radiation caries, trismus, and osteoradionecrosis).

The majority of patients (n = 15) developed OM within 10 days of commencing RT, and in the majority of patients (n = 9), it continued for two weeks after stopping RT, which is in accordance with the previously described results in the literature [9,10,11,12]. The largest number of patients developed third-degree OM; that is, they could only take liquid food. In this research, we used the World Health Organization scale for evaluating OM, which is based on the clinical findings and the ability to take food. Given that this is a retrospective study, the level of OM could be assessed only based on the possibility of food intake. 

The results of this study show that eight patients treated OM in line with the recommendations of an otorhinolaryngologist, and ten patients treated OM based on the recommendations of an oral medicine specialist. As far as we are aware, there is no research linking the treatment of OM to the recommendations of specialists. Additional research in this area and synergistic refinement of knowledge between the two professions based on training, such as congresses, symposia, and the writing of educational, professional scientific articles, contribute to a better understanding of the need for the dental treatment of oncology patients.

Most patients who developed OM used polyvinyl-pyrrolidone gel—Gelclair^®^—as a mitigating agent. The treatment of OM with polyvinyl-pyrrolidone adheres to the guidelines worldwide [13]. Its chemical composition is polyvinyl-pyrrolidone and hyaluronic acid. The gel forms a protective coating on the exposed nerve endings, creating a thin protective layer, which alleviates the sensation of pain caused by exposed nerve fibers [39]. Chin et al. [40], in their study, explained the multiple benefits of using a gel based on polyvinyl-pyrrolidone. In their study, patients receiving RT/CT were divided into two groups: the polyvinyl-pyrrolidone group and the placebo group. Grade III OM was more frequent and lasted almost 20 days longer in the placebo group. In the placebo group, five people developed oral candidiasis, while in the group that used this gel, only one person developed oral candidiasis, implying that the agent has a good antimicrobial effect.

In addition to Gelclair^®^, two patients reported the use of betamethasone ointment; baking soda solution; and a “cocktail” of antiseptics, anesthetics, and corticosteroids. Corticosteroid ointments, based on betamethasone, have an anti-inflammatory effect [41]. Normal saline or sodium bicarbonate (baking soda) provides relief from mild-to-moderate pain in OM, but according to MASCC/ISOO, neither one is effective enough [13]. The so-called “magic mouthwash”, i.e., solutions based on antiseptics, anesthetics, and corticosteroids, simultaneously relieves pain and provides protection against secondary infection from opportunistic microorganisms, but caution should be taken due to possible trauma when chewing, leading to numbness of the mucous membrane [42].

Potentially, one of the most important agents in the treatment and prevention of OM is benzydamine hydrochloride, (N, N-dimethyl-3-[(1-benzyl-1H-indazol-3-yl) oxy]-1-propanamine), a local anti-inflammatory drug with analgesic and anesthetic properties [43]. Kazemian et al. [44] stated in their research that the grade-three mucositis, according to the WHO scale, was 2.6 times more frequent in the group of patients who did not take benzydamine hydrochloride than in the group that did take it, and similar results were published by Rastogi et al. [45]. In the most recent guidelines, MASCC/ISOO recommends benzydamine for the prevention of OM in patients undergoing head and neck RT at radiation doses greater than 50 Gy (LoE I), and also for the prevention of OM in head and neck patients undergoing RT with simultaneous application of CT (LoE II) [9]. The patients in our study did not use benzydamine hydrochloride, but the results do not explain the reason for the choice or the options for the choice of therapy.

There are some issues which are important to state for this study. For 15 years, the dentists from the Department of Oral Medicine have been involved in the interdisciplinary dental management of patients irradiated in the head and neck area before, during, and after completed RT. There is no official protocol for the management of OM, as well as other side effects of RT within the Clinical Hospital Center Zagreb, but the MASCC protocol and guidelines are followed. The Department of Oral Medicine and Department of Otolaryngology are parts of the same Clinical Hospital Zagreb. The problem is that not all oncologists refer patients to a dentist so that they can receive adequate dental care and information about the side effects of RT. Unfortunately, to date, there is no official team which includes dentist as an equal member of the oncology team in the treatment of HNC patients; rather, it is more about the individual commitment and knowledge of the surgeon and/or oncologist who will refer the patient to a dental treatment. Furthermore, there are no medical records about the intensity and eventual management of OM, except in the case of patients referred to the Department of Oral Medicine. The results of this study, unfortunately, further confirm that dental care for patients irradiated in the head and neck area is still not adequate or satisfactory.

## 5. Conclusions

Although OM is the most difficult side effect to tolerate, our preliminary results suggest that the management of OM has not been given enough attention and that dentists are not adequately involved in the multidisciplinary team that treats patients with HNC. The treatment of OM with polyvinyl-pyrrolidone adheres to global guidelines. The reasons for the non-existence of other therapeutic approaches to OM (based on relevant guidelines) are not clear in regard to the majority of respondents. The application of other means and procedures mentioned in the discussion of this paper may depend on the capabilities of the patients and the institution where the patient is treated, as well as the knowledge of the therapist.

## Figures and Tables

**Table 1:** Inclusion and exclusion criteria of the participants.

Inclusion Criteria	Exclusion Criteria
Adult patients	Patients under 18 years of age
Diagnosis of squamous cell carcinoma of the head and neck	Terminal stage of the disease
Head and neck RT with or without CT	

**Table 2:** Demographic data and localization of oral cancer among respondents.

Sex	Absolute Frequencies	%	Cumulative %
Male	19	63.3	63.3
Female	11	36.7	100.0
Total	30	100.0	
Age			
30–39	2	6.7	6.7
40–49	1	3.3	10.0
50–59	10	33.3	43.3
60–69	9	30.0	73.3
70–79	7	23.3	96.6
80–89	1	3.3	100
Total	30	100.0	
What kind of cancer do you have/have you had?			
Oropharyngeal carcinoma	6	20.0	20.0
Tongue carcinoma	10	33.3	53.3
Floor of the mouth carcinoma	3	10.0	63.3
Tonsil carcinoma	3	10.0	73.3
Larynx carcinoma	3	10	83.3
Nasopharynx carcinoma	3	10	93.3
Glottis carcinoma	1	3.3	96.6
Maxillary sinus carcinoma	1	3.3	100.0
Total	30	100.0	

**Table 3:** Information about which specialist recognized the cancer, awareness of changes in the mouth, and the waiting time for the first specialist examination, as well as for radiotherapy.

Who Recognized That You Have Cancer?	Absolute Frequencies	%	Cumulative %
ENT specialist/oncologist	22	73.3	73.3
Doctor of dental medicine	6	20.0	93.3
General practitioner	2	6.7	100.0
Total	30	100.0	
How long were you aware of the possible changes/cancer before you consulted a doctor?			
Less than a month	8	26.7	26.7
A month	4	13.3	40.0
Two months	8	26.7	66.7
A couple of months	8	26.7	93.3
A year or longer than a year	2	6.7	100.0
Total	30	100.0	
How long did you wait for the first specialist examination after you noticed the change?			
Less than 7 days	5	16.7	16.7
7–14 days	19	63.3	80.0
A month	4	13.3	93.3
More than a month	2	6.7	100.0
Total	30	100.0	
If the treatment was radiotherapy, how long did you wait for it to start?			
A month	8	26.7	16.7
Two months	15	50.0	76.7
Longer than two months	7	23.3	100.0
Total	30	100.0	

**Table 4:** Data regarding dentist involvement in oncology team.

Were You Referred to a Dentist before Starting Head and Neck Radiotherapy?	Absolute Frequencies	%	Cumulative %
Yes	14	46.7	46.7
No	16	53.3	100.0
Total	30	100.0	
If so, who referred you to the dentist?			
No one referred me to the dentist	16	53.3	53.3
ENT specialist	14	46.7	100.0
Total	30	100.0	
Did you go to the dentist after the instruction?			
Yes	14	46.7	46.7
No	16	53.3	100.0
Total	30	100.0	
Has the doctor pointed out to you the consequences that radiotherapy can have on the oral cavity?			
Yes	22	73.3	73.3
No	8	26.7	100.0
Total	30	100.0	
What side effects did the doctor warn you about?			
All of the side effects	19	63.3	63.3
OM and dry mouth	1	3.3	66.7
Dry mouth	1	3.3	70.0
OM, dry mouth, and taste disorder	1	3.3	73.3
I wasn’t warned	8	26.7	100.0
Total	30	100.0	

**Table 5:** Frequency of radiation therapy, onset, and duration of OM.

How Many Times Have You Been Irradiated?	Absolute Frequencies	%	Cumulative %
29	1	3.3	3.3
30	17	56.7	60.0
32	5	16.7	76.7
33	3	10.0	86.7
34	3	10.0	96.7
35	1	3.3	100.0
Total	30	100.0	
Did you develop OM as a result of radiation?			
Yes	22	73.3	73.3
No	8	26.7	100.0
Total	30	100.0	
If one of the consequences of radiotherapy was OM, please state when it started			
Within 10 days from starting radiotherapy	15	68.2	68.2
More than 10 days from starting radiotherapy	5	22.7	90.9
I don’t know	2	9.1	100.0
Total	22	100.0	
If one of the consequences of radiotherapy was OM, please state how long it lasted			
Immediately after the end of radiation	1	4.5	4.5
One week	4	18.2	22.7
Two weeks	9	40.9	63.6
More than two weeks	4	18.2	81.8
Irradiation in progress	2	9.1	90.9
I don’t know	2	9.1	100.0
Total	22	100.0	

**Table 6:** Degree of OM.

Degree of OM (All Patients)	Absolute Frequencies	%	Cumulative %
I could only eat liquid food	14	63.3	63.6
I could eat solid and liquid food	4	18.2	81.8
I couldn’t eat normally (parenteral nutrition)	4	18.2	100.0
Total	22	100.0	
Degree of OM (patients receiving radiation with adjuvant chemotherapy).			
I could only eat liquid food	8	57.2	57.2
I could eat solid and liquid food	3	21.4	78.6
I couldn’t eat normally (parenteral nutrition)	3	21.4	100.0
Total	14	100.0	
Degree of OM (patient receiving radiation)			
I could only eat liquid food	5	62.5	62.5
I could eat solid and liquid food	1	12.5	75
I couldn’t eat normally (parenteral nutrition)	2	25	100.0
Total	8	100.0	

**Table 7:** Data regarding instructions and treatment of OM.

According to Whose Instructions Did You Treat OM?	Absolute Frequencies	%	Cumulative %
According to the instructions of the dentist/spec. oral medicine	10	45.4	45.4
According to the instructions of the doctor responsible for the underlying disease	8	36.4	81.8
No treatment	4	18.2	100.0
Total	22	100.0	
Please list what you have used to relieve the symptoms of OM			
Gelclair	7	31.8	31.8
Gelclair, sage tea	2	9.1	40.9
Sage tea	2	9.1	50.0
Mouthwash	1	4.5	54.5
Sage tea, chamomile	1	4.5	59.0
Gelclair, corticosteroids, and anesthetic “cocktails”	1	4.5	63.5
Gelclair, Anaftin	1	4.5	68
Gelclair, Beloderm, Sage tea, a solution of baking soda, a cocktail of antiseptics, anesthetics, and corticosteroids	2	9.1	77.1
Nothing	5	22.7	100.0
Total	22	100.0	

## Data Availability

Research data available on request from the authors.
